# TASTY trial: protocol for a study on the triad of nutrition, intestinal microbiota and rheumatoid arthritis

**DOI:** 10.1186/s12937-025-01089-6

**Published:** 2025-04-07

**Authors:** Sofia Charneca, Ana Hernando, Inês Almada-Correia, Joaquim Polido-Pereira, Adriana Vieira, Joana Sousa, Ana Santos Almeida, Carla Motta, Gonçalo Barreto, Kari K. Eklund, Ana Alonso-Pérez, Rodolfo Gómez, Francesco Cicci, Daniele Mauro, Salomé S. Pinho, João Eurico Fonseca, Patrícia Costa-Reis, Catarina Sousa Guerreiro

**Affiliations:** 1 Laboratório de Nutrição, Faculdade de Medicina, Universidade de Lisboa, Centro Académico de Medicina de Lisboa, Lisbon, Portugal; 2 Faculdade de Medicina, Universidade de Lisboa, Centro Académico de Medicina de Lisboa, Lisbon, Portugal; 3 GIMM - Gulbenkian Institute for Molecular Medicine, Lisbon, Portugal; 4 Serviço de Reumatologia, ULS Santa Maria, Centro Académico de Medicina de Lisboa, Lisbon, Portugal; 5 Instituto de Saúde Ambiental, Faculdade de Medicina, Universidade de Lisboa, Centro Académico de Medicina de Lisboa, Lisbon, Portugal; 6 Departamento de Alimentação e Nutrição, Instituto Nacional de Saúde Doutor Ricardo Jorge, Lisbon, Portugal; 7 Clinicum, Faculty of Medicine, University of Helsinki and Helsinki University Hospital, Helsinki, 00029 Finland; 8 Department of Rheumatology, Helsinki University Hospital and Helsinki University, Helsinki, Finland; 9 Musculoskeletal Pathology Group, Institute IDIS, Santiago University Clinical Hospital, 15706 Santiago de Compostela, Spain; 10 Dipartimento di Medicina di Precisione, Università Della Campania L. Vanvitelli, Naples, Italy; 11 Institute for Research and Innovation in Health (i3s), University of Porto, Porto, Portugal; 12 School of Medicine and Biomedical Sciences (ICBAS), University of Porto, Porto, Portugal; 13 Faculty of Medicine, University of Porto, Porto, Portugal; 14 Pediatric Rheumatology Unit, ULS Santa Maria, Lisbon, Portugal

**Keywords:** Mediterranean diet, Microbiota, Intestinal permeability, Endotoxemia, Rheumatoid arthritis

## Abstract

**Background:**

The gut microbiota has been implicated in the onset and progression of Rheumatoid Arthritis (RA). It has been proposed that gut dysbiosis impairs gut barrier function, leading to alterations in mucosal integrity and immunity. This disruption allows bacterial translocation, contributing to the perpetuation of the inflammatory process. Since diet is recognised as a key environmental factor influencing the gut microbiota, nutritional interventions targeting RA activity are currently being explored. This study aims to investigate whether a dietary intervention based on a typical Mediterranean Diet enriched with fermented foods (MedDiet +) can impact the gut microbiota, intestinal permeability, and RA-related outcomes.

**Methods:**

One hundred RA patients are being recruited at Unidade Local de Saúde (ULS) Santa Maria in Lisbon, Portugal, and randomly assigned to either the intervention (MedDiet +) or the control group. The 12-week nutritional intervention includes a personalised dietary plan following the MedDiet + pattern, along with educational resources, food basket deliveries, and clinical culinary workshops, all developed and monitored weekly by registered dietitians. The control group receives standardised general healthy diet recommendations at baseline. The intervention's effects will be assessed by evaluating disease activity, functional status, quality of life, intestinal permeability, endotoxemia, inflammatory biomarkers, intestinal and oral microbiota, serum proteomics, and serum glycome profile characterisation.

**Discussion:**

We anticipate obtaining integrative insights into the interplay between diet, the gut, and RA, while also exploring the underlying mechanisms driving these changes. This study, conducted by a multidisciplinary research team of registered dietitians, rheumatologists, biologists, and immunologists, aims to bridge the current gap between nutrition-related knowledge and RA.

**Trial registration:**

Registered in ClinicalTrials.gov (NCT06758817; date of registry: January 6th 2025).

**Supplementary Information:**

The online version contains supplementary material available at 10.1186/s12937-025-01089-6.

## Introduction

Rheumatoid Arthritis (RA) is a chronic immune-mediated disease characterised by inflammation of the synovial tissue of joints, which leads to progressive destruction of cartilage and bone, and ultimately impacts patients' functional capacity and quality of life [1, 2]. While genetic susceptibility is well-documented as a contributing factor in RA development, environmental factors are increasingly recognised to be involved in the onset and progression of the disease [3].

An imbalance in the composition and function of the gut microbiota, referred to as gut dysbiosis, has been identified as a key modulator of immune responses and an important factor in the development of immune-mediated disorders [4, 5]. The mechanism whereby this may occur is related to the influence of dysbiosis in disrupting the integrity and function of the intestinal barrier, as well as mucosal immunity [6, 7]. Increased intestinal permeability facilitates translocation of bacteria and their components, leading to endotoxemia, results in low-grade systemic inflammation, increased inflammatory biomarkers, and can thus contribute to the inflammatory process [7, 8]. The increased inflammation may take place not only at the epithelial level but also systemically, affecting the joints and linking intestinal dysfunction and rheumatic disorders [9]. Although dysbiosis and gut barrier dysfunction have been described in RA [10, 11], the specific ways in which diet could influence the relationship between gut integrity and systemic inflammation in RA remain to be fully elucidated.

Over the past few years, evidence has shown that patients with RA exhibit significant changes in their intestinal microbiota composition compared to healthy controls, including a marked decrease in α-diversity [12]. In light of these findings, the therapeutic modulation of the gut microbiota is being increasingly explored in RA, with a particular interest in dietary interventions, as diet is recognised as one of the main environmental factors influencing intestinal microbiota [13]. Both microbial and dietary metabolites have immunomodulatory properties that might be beneficial and could help manage immune-mediated and inflammatory disorders, including RA [14]. The relevance of whole dietary patterns, rather than individual nutrients, is progressively being addressed in RA, with an emphasis on the Mediterranean Diet (MedDiet) [6, 15, 16]. In this context, our group has recently shown that higher adherence to the MedDiet is associated with lower disease activity, lower impact of disease, and lower functional disability in RA patients [17].

It is also worth noting that the implications of the microbiota in RA go beyond the gut. Perturbations in oral microbiota have been found in individuals at high risk for RA [18], and in patients already diagnosed with RA [19]. Of interest, epidemiological evidence has linked periodontitis with RA, and it is hypothesised that certain perioral bacteria may contribute to the shift from health to disease by making post-translational modifications of proteins, such as citrullination, and thus provoking autoantibody production [20, 21]. In line with this, post-translational modification by glycosylation of circulating antibodies has been also described to precede the onset of other immune-mediated diseases such Inflammatory Bowel Disease [22]. This adds to the possible mechanisms contributing to RA onset and may also play a role in RA progression. Therefore, both oral and gut microbiota should be explored in integrative study designs.

Overall, when considering nutritional interventions in RA, the promotion of a symbiotic microbiota and its consequent influence on intestinal barrier integrity, endotoxemia and inflammatory biomarkers represents a plausible mechanism linking diet to improved clinical outcomes in these patients. Accordingly, this trial has been designed to investigate the triad of nutrition, intestinal microbiota, and RA by assessing the effects of a nutritional intervention specifically designed to enhance gut microbiota diversity.

## Methods

### Study design, setting and aims

This is a single-centre randomised controlled trial, coordinated and implemented at Faculdade de Medicina, Universidade de Lisboa (FMUL), Gulbenkian Institute for Molecular Medicine (GIMM) and Unidade Local de Saúde (ULS) Santa Maria, Centro Académico de Medicina de Lisboa (CAML), Lisbon, Portugal. This trial is registered in ClinicalTrials.gov (NCT06758817; date of registry: January 6th 2025). This trial was approved by the ethics committee of CAML (Ref. Nº114/22).

We aim to investigate whether a nutritional intervention based on a MedDiet enriched with fermented foods (MedDiet +) may influence disease activity, functional status, quality of life, intestinal permeability, endotoxemia, inflammatory biomarkers, intestinal and oral microbiota, serum proteomics and serum glycome.

The first participant was enrolled in July 2023, and data collection is anticipated to conclude by the end of 2025. Currently, the effect of the MedDiet on the Disease Activity Score in 28 joints (DAS28) of RA patients is not yet known, making it unfeasible to conduct a formal sample size calculation. Considering the number of potentially eligible RA patients treated at the Rheumatology Department at ULS Santa Maria, our recruitment capacity, available funding, and the size of recent published studies in this area, a convenience sample of 100 participants will be recruited.

Patients meeting eligibility criteria are randomly assigned to the intervention MedDiet + (*n* = 50) or control (*n* = 50) groups through an online block randomisation generator. Due to the nature of the trial, the participants and the registered dietitians responsible for implementing the study are not blinded. However, the clinicians evaluating the clinical outcome measures and the researchers performing the laboratory tests are blinded.

#### Primary endpoint

- Change of DAS28-ESR (Disease Activity Score in 28 joints calculated with erythrocyte sedimentation rate) from baseline to 12 Weeks.

#### Secondary endpoints

- Proportion of patients achieving:
  - ◦ European Alliance of Associations for Rheumatology (EULAR) moderate or good response
  - ◦ DAS28-ESR < 2.6
- Change in DAS28-CRP (Disease Activity Score in 28 joints calculated with C-reactive protein)
- Change on ultrasound score (32 joints scored 0-3 for grey scale and power Doppler)
- Proportion of patients who had a 10% improvement in ultrasound score
- Change in Short Form 36 Health Survey Questionnaire (SF36) results
- Change in Health Assessment Questionnaire (HAQ) results
- Changes in α- and β- diversity of the gut and oral microbiota
- Changes in the relative abundance of *Lactobacillus/Limosilactobacillus* and* Bifidobacterium* species in the gut
- Changes in butyrate-producing species in the gut microbiota
- Changes in H2S-producing species in the gut microbiota
- Change in the lactulose/mannitol ratio
- Change in Endotoxemia measured by TLR4 activation in reporter cells
- Change in inflammatory biomarkers (C-Reactive Protein, CPR; Erythrocyte sedimentation rate, ESR, faecal calprotectin)
- Change in serum soluble CD14 (CD14s), lipopolysaccharide-binding protein (LBP), and intestinal fatty acid binding protein levels (IFABP)
- Change in lipid profile (Triglycerides, Total Cholesterol, HDL and LDL Cholesterol)
- Change in body composition (Fat Mass, Fat-Free Mass, Total Body Water and Body Cell Mass)
- Change in anthropometric measurements (body mass index, BMI, and waist circumference)

We will also analyse zonulin levels, serum proteomics, and serum glycomics.

### Recruitment and eligibility criteria

**Fig. 1:**
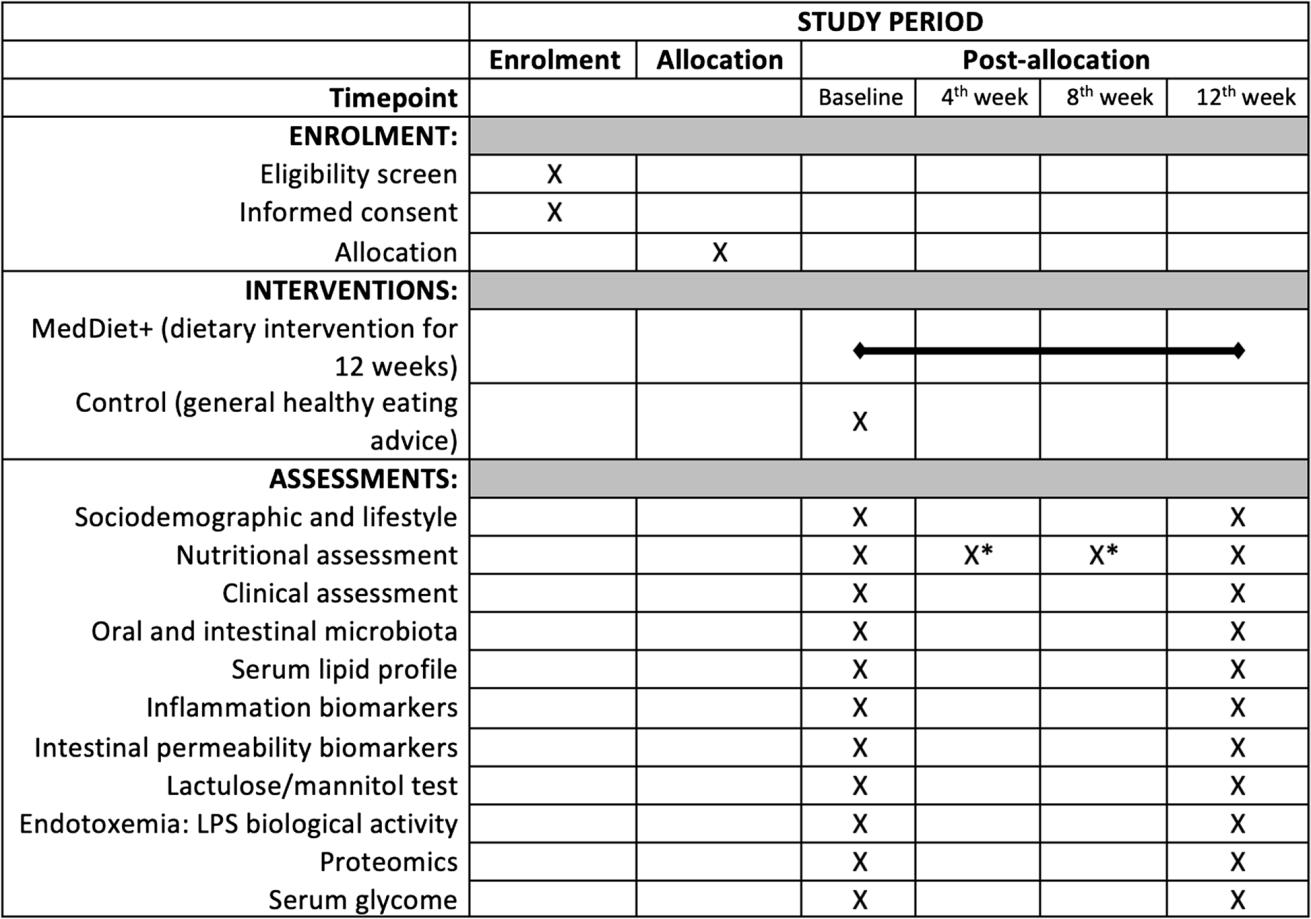
SPIRIT figure with standard protocol items of the TASTY trial. The SPRIT figure illustrates key components of the TASTY trial protocol and data collection time points from enrolment to the end of the 12 weeks. Abbreviations: Lipopolysaccharides (LPS). *Only in the MedDiet + Group

RA patients diagnosed according to the ACR/EULAR2010 criteria [23], aged 18 years old or older, are invited to participate in this study if eligibility criteria are met at their regular follow-up RA consultations. All participants sign a written informed consent form before any study procedures. Our inclusion criteria are diagnosis duration of at least one year, active disease (DAS28-ESR ≥ 2.6 units), stable medication for at least 12 weeks prior to the baseline assessment (including intra-articular steroid injections), and a low or medium adherence to the MedDiet (defined as a score of < 10 points in the 14-item tool by the PREvención com DIeta MEDiterránea trial, PREDIMED). Patients requiring therapeutic adjustments and/or intra-articular steroid injections during the trial are excluded. Our exclusion criteria are: antibiotic therapy within 4 weeks before enrolment; prednisolone dose > 7.5 mg/day; persistent use of non-steroidal anti-inflammatory drugs: diagnosis of inflammatory or irritable bowel disease, celiac disease, chronic diarrhoea; diagnosis of other immune-mediated or inflammatory diseases; major organ dysfunction; cancer diagnosed in the last five years; presence of health conditions which may impair the ability to consent to study participation (cognitive impairment/psychiatric disease). Regarding the use of antibiotics, although a general recommendation for a washout period is challenging to define, according to literature, a minimum of 4 weeks since cessation of antibiotics is recommended [24] and is considered in this trial. The overall diagram flow of the study is shown in the SPIRIT figure illustrated in Fig. 1. The SPIRIT checklist is provided as supplementary material.

### Interventions and comparators

#### MedDiet + group

The intervention is carried out for 12 weeks at ULS Santa Maria and includes an educational-based nutritional intervention with a structured nutrition plan prescribed by registered dietitians, educational food baskets, educational digital content, and clinical culinary workshops.

At baseline, a personalised nutritional plan is elaborated for each participant, and its implementation is closely monitored through weekly follow-up calls and monthly face-to-face appointments to fully involve participants and minimise dropout rates. To define an adjusted nutritional plan, nutritional requirements are individually assessed considering the patients' nutritional status and physical activity level. Energy, as well as protein, carbohydrate, and lipid requirements are calculated in agreement with the European Food Safety Authority (EFSA) dietary reference values [25]. The nutritional plan includes the number of food portions of each food group that participants must consume, in agreement with the MedDiet recommendations for the adult population [26]. To complement the traditional MedDiet, additional advice is provided to promote the consumption of specific fermented foods that potentially contain probiotic microorganisms, vitamins and bioactive compounds, such as fermented beverages, including kefir (provided for daily consumption) and kombucha (provided for consumption 2x/week). Foods with anti-inflammatory properties are also recommended, including sources of n-3 polyunsaturated fatty acids (PUFAs), such as oily fish, nuts and seeds, and a high amount of extra-virgin olive oil. The intake of vitamins, carotenoids, and phenolic compounds is promoted by the consumption of a wide variety of fruits, vegetables, legumes, whole grains, nuts and seeds, tea and infusions, herbs and spices, and extra-virgin olive oil. The nutritional plan can be adjusted if patients report issues with the consumption of specific foods.

Educational food baskets are delivered weekly with different typical ingredients of the MedDiet + pattern to encourage their inclusion in the patients' daily meals. This strategy ensures that every participant receives the same resources to meet the nutritional recommendations. We have secured a partnership agreement with a supermarket chain to overtake logistic aspects. Ingredients such as extra virgin olive oil, whole grains (whole grain bread, rice and pasta, oats), fresh fruits and vegetables, legumes, nuts and seeds, canned sardines and mackerels, herbs and spices, plain yoghurt, kefir, and kombucha are being provided.

Educational content consists of video recordings and two recipe books to share new ideas for cooking recipes, as well as educational and useful content to help participants meet the nutritional recommendations. It intends to empower patients with knowledge of different dishes and cooking methods to promote the inclusion of the foods provided in the food baskets. Recipes for other fermented foods, such as sauerkraut and sourdough bread are also included in the educational content. Finally, access to an online clinical culinary workshop is provided. The clinical culinary workshop allows for the application of the provided nutritional knowledge into culinary preparations through the demonstration of recipes and culinary methods.

#### Control group

At baseline, the control group receives a flyer with general recommendations on a healthy diet, based on the Portuguese brief guidance for healthy eating in primary health care [27]. No food baskets or any of the nutritional education strategies are implemented. To promote study adherence, a shopping voucher is given to each participant at the end of the trial. This comparator was chosen to reflect the standard care that could be given by clinicians.

### Data collection

#### Sociodemographic and lifestyle data

The participants' relevant clinical history is collected from their electronic hospital records (disease duration, current medication, and comorbidities). A structured questionnaire was developed to collect patients' age, sex, educational level, menopausal status, smoking status, and family history of rheumatic diseases.

#### Nutritional assessment

Nutritional assessment encompasses four components: nutritional intake, dietary pattern characterisation, body composition analysis, and anthropometric measurements. Nutritional intake is assessed by a 24-h dietary recall (24 h Recall), one of the most widely used tools in nutrition surveys to obtain detailed information about all food and beverages consumed in the past 24 h. A quantitative assessment with portion size quantification is included to provide a more comprehensive and detailed report. Adherence to the MedDiet is assessed with the Portuguese version [28] of the 14-Item Mediterranean Diet Assessment Tool developed by PREDIMED study authors [29, 30], in which a maximum score of 14 points can be achieved, being a strong adherence defined by a score ≥ 10 points. Anthropometric measurements (height, weight, and waist circumference) are performed by registered dietitians. BMI is calculated as weight/height squared (kg/m^2^) and bioelectrical impedance analysis (BIA) is performed to analyse body composition (Fat Mass, Fat-Free Mass, Total Body Water and Body Cell Mass). Nutritional assessment is performed at baseline and at the end of the trial for both groups and every month (baseline, 4th, 8th and 12th weeks) for the intervention group.

#### Clinical outcome measures

The DAS28-ESR and articular ultrasound examination of 28 joints are performed to assess disease activity. The DAS28 is the most widely used scoring system to determine disease activity in patients with RA [31, 32] considering the following items: number of tender and swollen joints in 28 joints (shoulders, elbows, wrists, metacarpophalangeal joints, hand proximal interphalangeal joints and knees); ESR (mm/hr), and a patient global health assessment based on a visual analogue scale (VAS-GH, range, 0–100). The DAS28-CRP is also being assessed as a secondary outcome measure. It encompasses the same items as the DAS28-ESR but substitutes the ESR with CRP in the formula.

Articular ultrasound examination is performed using a GE Logiq E9 equipment with a 6–15 MHz matrix linear probe. Doppler ultrasound (DUS) is used to assess the vascularisation of the synovial tissue. Ultrasound procedures and grey scale ultrasound (GSUS) and DUS grading are based on EULAR-OMERACT consensus [33–35]. The Doppler parameters are adjusted at the maximum sensitivity for slow flow (pulse repetition frequency of 0.4 kHz, lowest wall filter on 45 Hz, and 7.5 MHz Doppler frequency) with Doppler gain just below the noise level. In all patients, the wrists, metacarpophalangeal joints, and proximal interphalangeal joints are examined with ultrasound. Examinations are performed using a standardised dorsal and dorso-lateral scans. Erosions are also evaluated by GSUS as defined by the OMERACT consensus [36] and a semiquantitative score is used to evaluate ultrasound detected erosions [37].

As for patient-reported outcomes, functional status and quality of life are evaluated using the HAQ [38] and SF-36 short form [39] questionnaires, respectively. The HAQ assesses functional status through a disability index, and patient global and pain visual analogue scales [38]. The SF-36 includes a multi-item scale that assesses various health concepts such as limitations in physical activities related to health problems, limitations in social activities related to physical/emotional problems, limitations in day-to-day activities due to physical health problems, pain, mental health, limitations in the day-to-day activities because of emotional problems, vitality, and general health perceptions [39]. Clinical assessments are performed at baseline and after 12 weeks in both groups.

#### Oral and Intestinal Microbiota

Saliva samples are collected with a saliva collection kit while the faeces are self-collected by the participants with the OMNIgene GUT kit. Samples are processed according to the standard operating procedure, which defines biological samples' handling, processing, and freezing protocols to be preserved at the Biobank-GIMM, CAML.

For cell lysis, three bead beating cycles, in a total of 3 min of bead beating are included. We use the Ruptor Elite (OMNI International) with the following conditions: 1 min on at 6 m/s followed by 5 min rest. The bacterial DNA is extracted with the QIAamp PowerFecal Pro DNA Kit (Qiagen). Shotgun metagenomics sequencing will be performed, generating > 3 million reads per sample. The sequencing data resulting from the feces samples will be processed using the bioinformatics pipeline from the Microbiome in Health and Disease Translational Laboratory (GIMM), which includes quality filtering to remove low-quality reads and sequencing artefacts, removal of host DNA reads by mapping to the human genome (GRCh38), and elimination of optical duplicates. To perform taxonomic classification, a combination of k-mer and mapping approaches to a custom-developed reference database will be used, which includes a curated collection of bacterial and fungal genomes from publicly available databases (GTDB, UHGG). Similarly, for functional classification, a combination of k-mer and mapping approaches to publicly available functional databases such as Uniprot, KEGG, CAZy, and others will be used.

#### Lipid profile, inflammation and intestinal permeability biomarkers

Blood tests are performed to determine the serum levels of the lipid profile biomarkers (Triglycerides, Total Cholesterol, HDL and LDL Cholesterol) by standard operating procedures, as well as the serum levels of inflammatory and disease activity biomarkers (CRP and ESR). Although this study protocol encompasses the DAS28-ESR as a primary outcome, the serum CRP levels are also evaluated due to its high sensitivity for evaluating short-term inflammation [40]. Faecal calprotectin, an established marker of gut inflammation, is being analysed by a particle-enhanced turbidimetric immunoassay (CALiaGold© test).

Serum IFABP, lipopolysaccharide-binding protein (LBP) and sCD14 will be evaluated by Enzyme-Linked Immunosorbent Assay (ELISA), once data collection is completed. IFABP actively participates in dietary lipid metabolism by mediating fat absorption through binding and intracellular trafficking of free long-chain fatty acids [41]. Increased serum levels of IFABP, which is also a specific biomarker of gut epithelial integrity, have been shown in RA patients [42]. LBP, an acute-phase protein that carries the ability to bind to LPS [43], has been recognised as a sensitive serum biomarker for RA disease activity, as it significantly correlated with ESR, CRP, tender joint counts, swollen joint counts and DAS28 [44]. CD14s is also an acute phase protein whose hepatic production is increased in response to interleukin (IL)−6 in the setting of inflammation. CD14 stimulates the production of other pro-inflammatory cytokines, including IL-6, in an amplification loop that participates in RA pathogenesis [45]. Furthermore, CD14s has been shown to be increased in RA patients and correlate with DAS28 score and response to treatment [46]. These biomarkers, IFABP (serum diluted 1:4), LBP (serum diluted 1:500), and CD14s (serum diluted 1:15), will be measured from patient serum by sandwich ELISA (LBP cat. DY870; FABP2 cat. DY3078; CD14 cat. DY883; R&D Systems, USA), following the manufacturer instructions. Horseradish peroxidase conjugate will be detected with enhanced chemiluminescence substrate (cat. 32,106, Pierce, USA) and measured with a microplate reader (BMG Pherastar FS, BMG LABTECH, Germany).

Zonulin, a strong modulator of intestinal intercellular tight junctions, is recognised to play a role in the translocation of macromolecules and, consequently, in the tolerance/immune response balance [47]. Higher serum zonulin levels were shown to be accompanied by increased intestinal permeability and, in turn, the disruption of the intestinal barrier function has been proven to occur before the onset of the inflammatory phase of murine and human arthritis [48]. Zonulin (serum diluted 1:10) will be measured from patient serum by sandwich enzyme-linked immunosorbent assay (ELISA) (cat. E-EL-H5560, Elabscience, USA), according to the manufacturer instructions. Optical density will be measured with the microplate reader (BMG Pherastar FS, BMG LABTECH, Germany) set to read the absorbance at 450 nm with a wavelength correction set at 540 nm.

All ELISA assays will be performed in High-Throughput Screening (HTS) mode with reagent and liquid dispensing by the dispenser (CERTUS FLEX Fritz Gyger AG, Germany), serum sample dispensing by dispenser (ECHO 650, Labcyte, US) or manually, and washes by plater washer (EL406, Agilent BioTek, USA). These assays will be performed at the Finnish Institute for Molecular Medicine Finland (FIMM) High Throughput Biomedicine unit.

#### Intestinal permeability: lactulose/mannitol test

Intestinal Permeability is assessed through the lactulose/mannitol (Lac/Man) test, a gold standard for functional measurement of intestinal permeability in humans. Specific dietary recommendations for the 24 h before the test are provided to standardise conditions across participants and reduce variability in results. Although there is no consensus for dietary restrictions in this test, patients are advised to avoid some foods and beverages potentially containing lactulose and/or mannitol [49–51], such as dairy products, sweet potato, mushrooms, cauliflower, butternut squash, celery, peas, green beans, chicory, fennel, kimchi, sauerkraut, peach, watermelon, gelatin, gummies, candies, chewing gum, sauces, ‘diet’ and ‘light’ food products, artificial sweeteners, dietary supplements, soft drinks, and flavoured and alcoholic beverages.

After an overnight fast (≥ 8 h), patients are given a solution containing 5 g of lactulose and 1 g of mannitol and are encouraged to drink 1.5 L of water for 4 h and collect all the urine produced during that time. Lactulose and mannitol concentrations are determined by ultra-performance liquid chromatography-tandem mass spectrometry (UPLC-MS/MS). The lactulose and mannitol quantification is performed at Instituto Nacional de Saúde Dr. Ricardo Jorge in Lisbon, Portugal. UPLC-MS/MS and mass spectrometer conditions are further detailed in the supplementary material.

#### Endotoxemia: LPS biological activity

Toll-like receptor 4 (TLR 4) activation reflecting LPS bioactivity will be measured, when recruitment has finished and all samples have been collected, with HEK-Blue hTLR4 reporter cells (InvivoGen) engineered to produce secreted alkaline phosphatase in response to TLR4 stimulation. The method has been established and validated for cell-based High Throughput Screening, making it appropriate for population cohort sample screening [52]. Briefly, 70,000 cells/well will be seeded on a 384-well plate with 25 μl of cell culture media. Serum samples (2.5ul) will be added in duplicates to wells to obtain a final working concentration of 1% (v/v) per well. To determine whether TLR4 activation is due to LPS, we will add another set of sample duplicates with 0.1 mg/ml of polymyxin B (InvivoGen), an LPS inhibitor. Human AB serum 1% (v/v, Sigma-Aldrich) will serve as a negative control. A set of standard dilutions will be created with LPS-B5 Ultrapure from *Eschericia coli* O55:B5 (InvivoGen) diluted in endotoxin-free water with 1% Human AB serum. After incubation at 37° C for 24 h, we will add 10ul/well SEAP substrate QUANTI-BlueTM Solution (InvivoGen). Following a 6 h incubation at 37 °C, absorbance will be measured with a microplate reader (BMG Pherastar FS, BMG LABTECH, Germany). TLR4 activation due to LPS will be then determined by subtracting the activity remaining in the presence of polymyxin B from the total TLR4 activation measured. A linear standard curve will be plotted. The LPS biological activity will be performed at the Helsinki University (Finland).

#### Proteomics

Nowadays, various proteomic techniques are being applied to different biological samples, from both RA patients and experimental animal models, showing promise in identifying novel biomarkers and treatment targets [53]. In our trial, once all samples have been collected, blood serum samples will be studied through micro-liquid chromatography-mass spectrometry (micro-LC–MS/MS) using a hybrid quadrupole TripleTOF 6600 (Sciex, CA, USA). The proteomes will be identified through the qualitative shotgun data-dependent acquisition (DDA) method [54, 55], and protein levels will be measured through the quantitative sequential window acquisition of all theoretical mass spectra (SWATH) methods. A 5% false discovery rate (FDR) and a p-value ≤ 0.05 will be used to filter the dataset. FunRich software 3.1.3 will be used to determine proteome enrichment.

#### Serum Glycome characterisation

When patient recruitment has been concluded, serum will be processed and ultra high performance liquid chromatography (UHPLC) analysis will be performed using hydrophilic interaction liquid chromatography (HILIC) chromatography coupled with fluorescence detection and online electrospray MS for total serum glycome characterisation. From this analysis we will be able to identify unique signatures related with plasma proteins N-glycans alterations that can be associated with dietary intervention. Then, and given the fact that Fc glycosylation of IgG/IgA is a key factor for the definition of effector (inflammatory) function of IgG/IgA, we will characterise the antibody specific Fc glycosylation of IgG/IgA at the different time points before and after dietary intervention, by advanced glycoproteomics nano-liquid chromatography-electrospray ionization-tandem mass spectrometry (nanoLC-ESI–MS). The structural characterisation of the IgG Fc-N-glycans in terms of composition and abundance will be performed by advanced liquid chromatography coupled to mass spectrometry), that enables high-throughput analysis of IgG Fc-glycans in a subclass-specific manner.

**Fig. 2:**
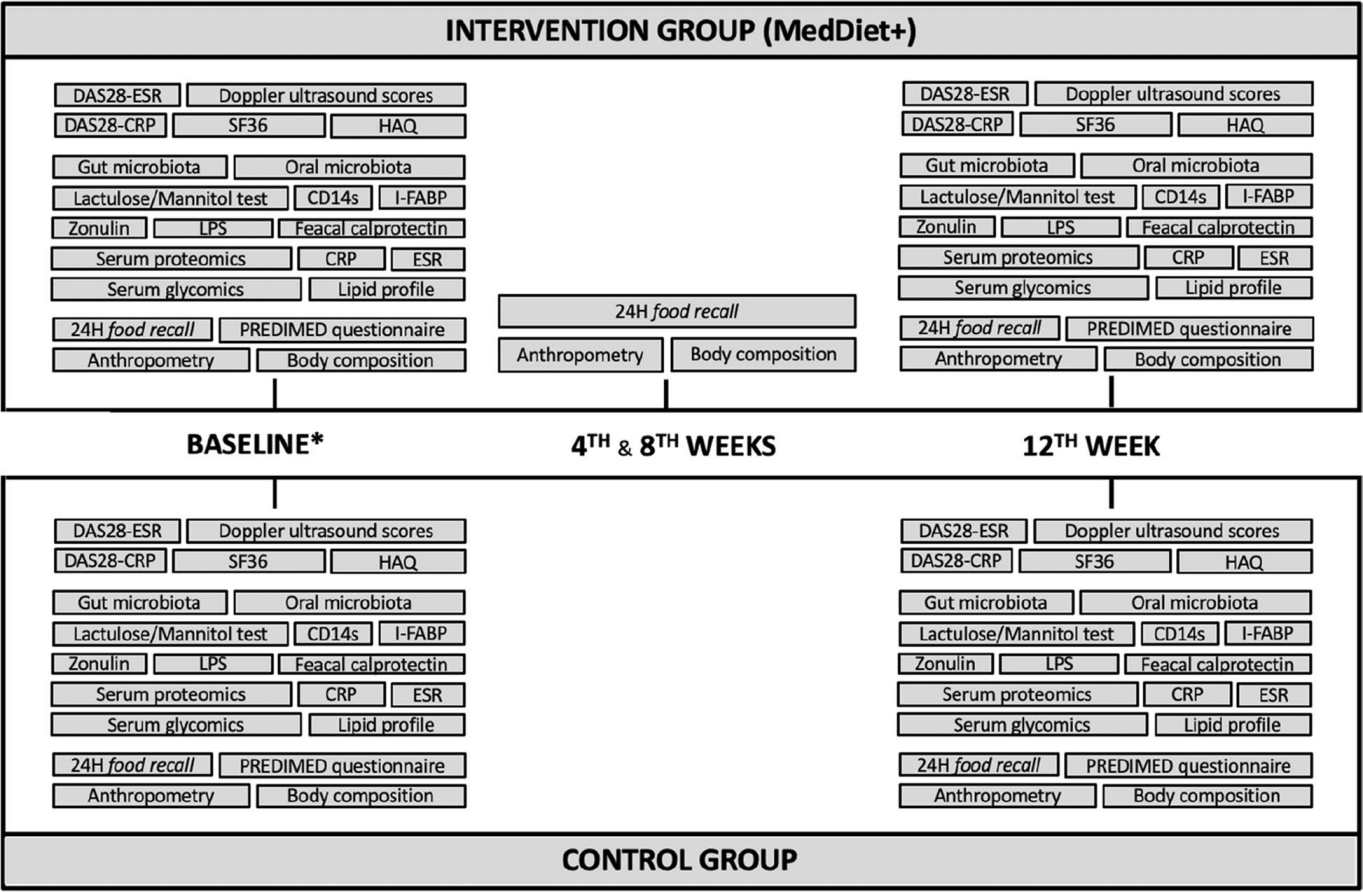
– Overview of the data collection throughout the trial. Visual representation of the specific variables to be collected in each phase of the study, which are identical at baseline and at the end of the trial (12th week) for both groups. Only the intervention group (MedDiet +) has in-person follow-up consultations with nutritional assessment at 4th and 8th weeks. *Sociodemographic and lifestyle data are also collected at baseline. Abbreviations: CRP: C-reactive protein; DAS28: Disease Activity Score using 28 joint counts; ESR: Erythrocyte sedimentation rate; HAQ: Health Assessment Questionnaire; I-FABP: Intestinal-fatty acid binding protein; LPS: Lipopolysaccharides; PREDIMED: Prevención con Dieta Mediterránea; SF36: 36-Item Short Form Health Survey

Figure 2 resumes all variables collected during the study timeframe.

#### Data management and statistical analysis

Collected data files in paper format are being securely stored in locked filing cabinets within the Nutrition Lab, FMUL, CAML, accessible only to authorized team members. All recorded data and collected samples are coded, and analysis will be performed anonymously. Only members of the research team will have access to data and samples. FMUL has the property of the resulting data and the right to disclose the results as merged data analysis. The Nutrition Lab of FMUL will have access to the final trial dataset and will be responsible for storing conserved data for five years after the last publication.

Statistical analysis will be conducted using SPSS (SPSS® Inc., Chicago, IL) version 28.0 or a subsequent updated version. The Normal data distribution will be verified using the Kolmogorov–Smirnov tests, considering the sample size. Depending on the normality of the data, parametric or non-parametric tests will be used. A *p*-value of less than 0.05 will be considered statistically significant. Descriptive statistics will be used to describe all variables, both by groups (intervention vs. control) and by timing (baseline and final visits). Characteristics between groups at baseline will be compared to identify significant differences that may confound results. To access differences between the baseline and the end of the intervention (paired sample test) as well as between groups (independent samples test), the student's T-Test or its non-parametric equivalent will be used. Multiple regression analysis will be conducted to address the influence of baseline characteristics. An analysis of covariance (ANCOVA) will be carried out when baseline differences need to be controlled, being the group (intervention/control) the independent variable, and the covariates the baseline characteristics and demographic data. To test how the MedDiet + pattern influences the study endpoints, a multivariate analysis of covariance (MANCOVA) will be conducted to assess group differences with multiple dependent variables and adjusting for confounding factors. The existence of a control group allows for statistical comparisons between groups and ensures the true effectiveness of the MedDiet + when adjusting for potential confounders.

## Discussion

Treat-to-target strategies have undoubtedly changed the paradigm of RA disease control and progression, significantly improving patient-related outcomes. However, despite the success of these novel treatment strategies, there are still important unmet needs for more effective treatment of RA. As full remission is challenging to achieve and sustain, both patients and clinicians are continuously looking for adjuvant therapies that may contribute to improving patients' overall well-being and quality of life. This study aims to investigate whether a nutritional intervention based on a MedDiet style pattern, enriched with fermented foods, can modulate the gut microbiota and, as a result, influence disease activity and inflammatory biomarkers. With this approach, we expect to obtain a synergistic benefit from the various dietary elements, with a potential impact on both microbiota modulation and RA disease activity.

Over the last few years, intestinal barrier dysfunction has been implicated as a relevant player in the pathophysiology of arthritis [42, 56]. Notably, the disruption of the gut integrity was shown to be reversible in RA [10], as effective biological disease-modifying antirheumatic drugs (bDMARDs) were shown to decrease gut permeability biomarkers. In fact, RA patients exhibited altered colonic tight junction proteins, as well as increased serum biomarkers of intestinal permeability [10]. A clinical response to bDMARDs in these patients correlated with a decrease in gut permeability markers [10]. These findings pave the way for the rationale that enhancing the gut barrier function may be a valuable mechanism for managing RA. The gut microbiota plays a crucial role in maintaining gut barrier integrity, as gut microbial metabolites, produced close to the gut epithelium, exert effects on both gut barrier function and immune responses [57]. Since the impairment of gut barrier function seems to be driven by dysbiosis [58, 59], interventions targeting the gut microbiota are of particular interest.

Several trials have shown that the MedDiet can positively influence gut microbiota composition [60–62]. For instance, Meslier et al. [60] found that a MedDiet intervention resulted in microbiome changes, including increased gene richness in individuals who achieved reduced systemic inflammation over the intervention (evaluated by serum high sensitivity CRP). A rise in the fibre-degrading *Faecalibacterium prausnitzii,* and a decrease in the potentially proinflammatory *Ruminococcus gnavus* were also observed [60]. Additionally, according to results by Ghosh et al. [61], adherence to the MedDiet was associated with an increased abundance of specific taxa. which negatively correlated with inflammatory markers, including CRP and IL-17 [61]. Of interest, the operational taxonomic units that showed a positive increase in the intervention cohort of this trial (compared with the non­intervention group) included species like *Faecalibacterium prausnitzii*, *Eubacterium* and *Roseburia* [61]. In the TASTY trial, enriching the traditional MedDiet with fermented foods is expected to further enhance gut microbiota modulation and diversity. On this subject, probiotics have also shown some degree of benefit in both animal models of arthritis and human studies, and fermented foods and beverages are highlighted as possible alternatives to probiotic supplements [63]. As for intestinal inflammation and permeability biomarkers, increased adherence to the MedDiet has been associated with a reduction in faecal calprotectin levels in healthy subjects [64] and a decrease in serum zonulin and endotoxin levels, along with other biomarkers of oxidative stress and inflammation, in patients with non-alcoholic fatty liver disease [65]. In RA, a systematic review of human prospective studies reported beneficial effects of the MedDiet in reducing pain and improving patients' physical function [66].

Although both intestinal and oral microbiota appear to be disturbed in RA patients, they seem to exhibit distinct behaviours. A systematic review [67] reported that the α-diversity of the microbiota was either decreased or unchanged in the gut of RA patients but increased or unchanged in the oral cavity. As oral and intestinal microbiota seem to follow distinct patterns, it is important to access both. Recent trials have shown relevant associations between what we eat and oral microbiota composition. Using 16S rRNA amplification sequencing, associations were found in specific nutrient intake with oral microbial community diversity and richness [68]. Furthermore, a recent systematic review reported that sugar‐rich diets have a significantly unfavorable effect on the diversity and balance of the oral microbiota [69]. Interestingly, following the MedDiet has been shown to reduce levels of periodontopathogenic bacteria in saliva and, thus, may be a promising dietary strategy for maintaining oral homeostasis [70]. Even though oral dysbiosis is gaining more attention in RA research, the effects of a nutritional intervention on its modulation remain largely unknown.

The MedDiet is also recognised for its anti-inflammatory and antioxidant properties, attributed to specific components, including vitamins, carotenoids, and phenolic compounds [71]. This dietary pattern also promotes a high intake of foods rich in PUFAs, which promote a balanced n-6:n-3 fatty acid ratio, and a high intake of extra-virgin olive oil, which is rich in monounsaturated fatty acids (MUFAs), influencing the expression of pro-inflammatory genes, and the activity of immune cells [71]. Another relevant characteristic of the MedDiet is its richness in dietary fibre, which enhances microbial fermentation and the production of active metabolites such as Short Chain Fatty Acids (SCFA) in the gut, positively impacting intestinal function and integrity [16, 72]. On this subject, butyrate has been shown to significantly reduce bacterial translocation in the gut, underlining the relevance of a symbiotic microbiota in maintaining the epithelial barrier function [73]. In individuals at increased risk of RA, higher serum levels of SCFA, particularly butyrate and acetate, were shown to be associated with non­progression to arthritis [74]. Overall, the production of SCFA is among the possible pathways linking gut microbiota dysbiosis with RA progression, together with other microbiome-derived metabolites, molecular mimicry, microbiome-induced intestinal immune responses, and intestinal epithelial cell autophagy [75]. Collectively, these dietary compounds are expected to synergistically contribute to the effects of the nutritional intervention.

The TASTY trial stands out for its innovative intervention, combining a well-established health-promoting dietary pattern with specific components designed to promote beneficial microbiota modulation. This trial also includes a dynamic set of educational resources to improve literacy, compliance, and empower participants to make better food choices. Another differentiating characteristic of the TASTY trial is its extensive data collection and comprehensive biomarker analysis.

Another strength of the TASTY study design is that the dietary intervention goes beyond merely providing a dietary plan, addressing a key limitation in previous dietary trials: participants' difficulty in adhering to recommendations. Several nutritional education strategies are implemented based on the "Clinical Culinary" concept to ease the process of changing eating habits and maximise the effects of the nutritional intervention. Clinical Culinary is a new, evidence-based component of clinical care that incorporates knowledge of nutrition science, including food and cooking, into current medical disease prevention and treatment [76]. The main goal of this approach is to create a positive behavioural change by equipping patients with the knowledge and practical skills needed to improve their health. The systematic delivery of food baskets will also help ensure standardised access to the recommended foods, which is crucial to make sure participants from different financial backgrounds have the means to successfully implement the nutritional intervention. Additionally, the content of the food baskets provided to participants will carefully be selected by the registered dietitians according to the MedDiet principles, meaning that minimally processed foods will be chosen. For instance, plain yoghurt will be provided to minimise participants' consumption of food additives, such as artificial sweeteners, due to their reported effects on gut microbiota [77]. This trial will also allow us to address if significant differences in dietary intake and clinical outcomes may be achieved by standard care that could be given by clinicians (control group) and to which degree a structured nutritional plan developed by registered dietitians (MedDiet +) implies additional benefits. Weaknesses of this trial include the inability to observe long-term effects beyond the intervention period and the reliance on self-reported food intake, which is susceptible to inaccuracies. However, the close follow-up during the intervention period, along with the educational and patient empowerment strategies, were designed to mitigate these limitations. Regarding eligibility criteria, the exclusion of antibiotics for only four weeks before baseline may represent an additional limitation, as different treatment schemes can impact gut microbiota in distinct ways. The class of antibiotics, the dosage, the route of administration, and the characteristics of the individual are some of the reported mechanisms that justify the varying disturbance caused by antibiotics in the microbial communities of the gut [78].

We believe that the results of this trial will contribute to identifying potential targets for both therapeutic and preventive approaches in RA, due to the deep exploration of inflammation and mucosal microbial sites. The extensive data collection may contribute to the development of personalised treatments and change the paradigm of RA treatment. Our study has the potential to provide novel strategies to control RA activity. The results of this trial can contribute to establish strong scientific foundations for the use of diet interventions as an adjuvant therapy in RA, as a means to intercept the perpetuation of inflammation, prevent disease progression and irreversible damage that deteriorates patients' quality of life, which is particularly important in life-long conditions like RA.

## Conclusion

The TASTY trial aims to bridge the existing gap between nutrition-related knowledge and disease pathogenesis and activity in RA. A multidisciplinary research team was established, including registered dietitians, rheumatologists, biologists, and immunologists to evaluate this nutritional therapy's impact on intestinal function and overall RA disease activity.

## Supplementary Information

Supplementary Material 1.

## Data Availability

No datasets were generated or analysed during the current study.

## Abbreviations

bDMARDs: Biological disease-modifying antirheumatic drugs
BMI: Body mass index
CAML: Centro Académico de Medicina de Lisboa
CRP: C-Reactive Protein
DDA: Data-dependent acquisition
DAS28: Disease Activity Score using 28 joint counts
DAS28-CRP: Disease Activity Score in 28 joints calculated with C-reactive protein
DAS28-ESR: Disease Activity Score in 28 joints calculated with erythrocyte sedimentation rate
DUS: Doppler ultrasound
ELISA: Enzyme-Linked Immunosorbent Assay
ESR: Erythrocyte sedimentation rate
EFSA: European Food Safety Authority
FMUL: Faculdade de Medicina, Universidade de Lisboa
FIMM: Finnish Institute for Molecular Medicine Finland
GSUS: Grey scale ultrasound
GIMM: Gulbenkian Institute for Molecular Medicine
HAQ: Health Assessment Questionnaire
IFABP: Intestinal fatty acid binding protein levels
IL: Interleukin
Lac/Man: Lactulose/mannitol
LBP: Lipopolysaccharide-binding protein
LPS: Lipopolysaccharides
MedDiet: Mediterranean Diet
MedDiet +: Mediterranean Diet enriched with fermented foods
micro-LC–MS/MS: Micro-liquid chromatography-mass spectrometry
Nano-LC-MS: Nano-liquid chromatography-electrospray ionization-tandem mass spectrometry
PUFAs: Polyunsaturated fatty acids
PREDIMED: PREvención con DIeta MEDiterránea
RA: Rheumatoid Arthritis
CD14s: Serum soluble CD14
SCFA: Short Chain Fatty Acids
SF36: Short Form 36 Health Survey Questionnaire
SWATH: Sequential window acquisition of all theoretical mass spectra
TLR4: Toll-like receptor 4
UPLC-MS/MS: Ultra-performance liquid chromatography-tandem mass spectrometry
ULS: Unidade Local de Saúde
VAS: Visual analogue scale
